# Comparison of functional outcome after intracerebral hemorrhage in patients with or without end stage renal disease on hemodialysis: a propensity-score matched study

**DOI:** 10.1186/s12883-024-03932-5

**Published:** 2024-11-18

**Authors:** Kotaro Tsutsumi, Matthew Nguyen, Victoria Nguyen, Zhu Zhu, Mohammad Shafie, Jay Shah, Masaki Nagamine, Dana Stradling, Diana Dench, Wengui Yu

**Affiliations:** 1 Department of Neurology, University of California, Irvine, 200 S. Manchester Ave. Suite 206, Orange, CA 92868 USA; 2 School of Medicine, University of California, Irvine, Orange, CA USA; 3 Department of Neurology, University of Kentucky, Lexington, KY USA

**Keywords:** Intracerebral hemorrhage, End stage renal disease, Hemodialysis, Mortality, Obesity

## Abstract

**Background:**

End stage renal disease (ESRD) requiring hemodialysis (HD) increases mortality among patients with intracerebral hemorrhage (ICH). The aim of this study is to investigate the clinical characteristics and outcome of ICH patients with ESRD on HD versus propensity-score matched controls.

**Methods:**

This is a single center retrospective study. Consecutive ICH admissions at the University of California, Irvine Medical Center from January 1, 2018 to July 31, 2022 were analyzed.

**Results:**

Among 347 ICH admissions that met inclusion criteria, 24 patients (6.92%) had ESRD on HD. Compared to patients without ESRD, patients with ESRD on HD had significantly higher rate of diabetes mellitus (79.2% vs. 36.8%, *p* < 0.01) and in-hospital mortality (25% vs. 7.43%, *p* < 0.01). There were no significant differences in demographics, other comorbidities, clinical characteristics, good (mRS score 0–3) or poor (mRS score 4–5) functional outcomes, rate of comfort care and the time to comfort care decision between the 2 groups. After propensity score matching, the ESRD group had a significantly higher in-hospital mortality rate (27.3% vs. 8%, *p* = 0.012) and a lower rate of obesity (9.1% vs. 34.1%, *p* = 0.02). Among patients who died during admission, ESRD on HD status did not inadvertently influence end-of-life care decisions. Univariate logistic regression and area under curve analysis showed that ICH score ≥ 3 was a predictor of increased mortality in both ESRD and non-ESRD groups.

**Conclusions:**

ICH patients with ESRD on HD had significantly higher in-hospital mortality and lower rate of obesity than propensity score matched controls, suggesting a survival benefit from obesity. ICH score ≥ 3 is an independent predictor for poor outcomes in both ESRD and non-ESRD groups.

## Introduction

End-stage renal disease (ESRD) is a debilitating condition and has emerged as a leading cause of mortality globally [1]. A common treatment for ESRD is hemodialysis (HD), which, while improving survival rates and quality of life, may lead to cardiovascular and neurological complications, including labile blood pressure and intracerebral hemorrhage (ICH) [2, 3]. 

Hypertension (HTN) is the strongest cardiovascular risk factor for ESRD. In a large, population-based study, treatment-resistant HTN were found to be associated with significantly increased risk for ESRD [4]. Hypertension is also the major cause of spontaneous ICH [5]. In our previous study, 31.6% of patients with spontaneous ICH were found to have resistant HTN [6]. 

In patient with ESRD on HD, heightened blood pressure variability and the use of anticoagulation during HD increase the risks of ICH and poor functional outcome after ICH [7, 8]. A few previous studies showed that ICH patients with ESRD on HD had much higher mortality rate than those without ESRD (40-67.3% versus 10–21%) [7–10]. Although numerous variables, including age, Glasgow Coma Scale (GCS) score, hematoma volume, the presence of intraventricular hemorrhage (IVH), lack of antihypertensive drug use, and treatment modality, were reported to be associated with increased mortality, the major causes of death in ICH patients with ESRD remains unclear. The aim of this study is to investigate the clinical characteristics and outcome prognosticators of mortality in ICH patients with ESRD on HD versus propensity-score matched controls.

## Methods

### Data availability and ethical approval

This study was approved by our Institutional Review Board (IRB) and the Ethics Committee. Our research protocol was reviewed by the IRB and Human Research Protections Committee, University of California, Irvine Office of Research. This retrospective study involves minimal risk to study subjects and was approved in Exempt Category. The IRB approval ID is HS# 2018–4332. All methods in the study were performed in accordance with the relevant guidelines and regulations. Any anonymized data and syntax of statistical analyses will be made available from the corresponding author upon reasonable request.

### Study design and study population

This is a retrospective single center study and followed the STROBE reporting guideline for observational studies [11]. Consecutive patients with spontaneous ICH at the University of California Irvine Medical Center between January 1, 2018 and July 31, 2022 were identified by searching the Vizient clinical database using the International Classification of Diseases, 10th revision (ICD-10) code for ICH (I61) as a primary or secondary discharge diagnosis. Vizient contains data from our stroke center and over 97% of US academic medical centers [ 12]. The following information was abstracted from the Vizient database and independent chart review from our electronic medical record system EPIC: age, gender, race, past medical history, recreational drug use, urine drug screen, the highest systolic blood pressure (SBP) in the Emergency Room, initial GCS score, home medications, ICH location, IVH, ICH score, length of stay (LOS) in the intensive care unit (ICU) and in the hospital, and modified Rankin Scale (mRS) score at hospital discharge. The data on history of HTN, diabetes mellitus (DM), hyperlipidemia, tobacco use, and alcohol use were all directly extracted from the chart’s medical history section along with confirmation via examining patients’ hospital admission notes. Obesity was defined as body mass index of 30 or greater. Methamphetamine and cocaine use were evaluated via urine toxicology screen.

ICH location was categorized into subcortical (basal ganglia or thalamus), cortical, brainstem, cerebellum, or IVH [6]. ICH score was calculated as previously described [13]. Functional outcome was estimated using mRS score. Since patients with initial poor outcome (mRS 4–5) could improve significantly by one year after ICH, functional outcomes were categorized to good (mRS 0–3), poor (mRS 4–5), or death (mRS 6) at hospital discharge [14]. ICU LOS was binarized with a cutoff of 21 days per prior studies [15, 16]. ICH score was binarized with score of 3 as a threshold [17]. 

ICH from cerebral aneurysm, arteriovenous malformation, brain tumors, coagulopathy, or traumatic brain injury were excluded from the study. Patients with subdural hemorrhage, epidural hemorrhage, or missing data on functional outcomes were also excluded.

All ICH patients were managed initially in the dedicated Neuroscience Intensive Care Unit (ICU) and then Stroke Stepdown Unit with standard ICH order-set and clinical pathway by board-certified neurosurgeons, neurointensivists and vascular neurologists per AHA/ASA guidelines [18]. 

### Statistical analysis

Clinically relevant variables and outcome measures were identified and compared between ESRD and non-ESRD groups via the chi-squared test. Propensity score matching of factors reported to be associated with ESRD was performed with match tolerance of 0.1 and match ratio 4:1 for the 2 group patients. Sensitivity analysis was done after propensity score matching to investigate the prognosticators of increased mortality in ICH patients with ESRD on HD. Association of individual variables with mortality was evaluated via univariate logistic regression. Multivariate logistic regression analysis was not conducted given that the only significant variables among the univariate analysis were ICH score and IVH, along with our relatively low sample size in the ESRD group. To assess the predictive capacity of ICH score in both ESRD and non-ESRD groups, we constructed receiver operating characteristic (ROC) curves and calculated their area under the curve (AUC). Bootstrapping was employed to calculate confidence intervals for each individual evaluation metric. The statistical analyses were performed using SPSS statistical software (IBM, SPSS Inc.) and Python programming language. A significance level of 0.05 was used for all tests.

## Results

### Clinical characteristics

Of the 453 consecutive patients screened, 347 met inclusion criteria. The average age of study patients was 64.4 ± 15.6 and 61.4% of them were male. In the entire cohort, 90.8% of the patients had HTN as compared to 39.8% with DM, 37.2% with hyperlipidemia, and 22.8% with obesity. 58.5% of the patients had SBP greater than 180 at initial presentation. The most common ICH location was subcortical (52.2%). The average ICH score was 1.57 ± 1.45. The in-hospital mortality rate was 8.65%.

Of the 347 study patients, 24 (6.92%) patients had ESRD and were treated with HD (Table 1). There were two patients with ESRD who were not on HD, and therefore were not included in the ESRD on HD group. None of the ESRD patients were on peritoneal dialysis or home hemodialysis at the time of presentation to the hospital for ICH. The average age of the ICH patients with ESRD on HD was 58.6 ± 10.7 and 60.0% of them were male. All of them (100%) had HTN, 79.2% had DM, 12.5% had atrial fibrillation, 16.7% of patients were on anticoagulation, and 66.7% had SBP above 180 at initial presentation. Among the 20 patients with information on dialysis vintage, the average years the patients had been on HD was 4.6 ± 4.3. Due to small sample size, we were unable to analyze the effect of longer dialysis vintage on mortality. Among the ICH patients with ESRD on HD, the most common ICH location was subcortical (45.8%), with an average ICH score of 1.50 ± 1.50, and in-hospital mortality rate of 25%. Compared to the ICH patients without ESRD, the patients with ESRD on HD had significantly higher rates of DM (79.2% vs. 36.8%, *p* < 0.01) and in-hospital mortality (25% vs. 7.43%, *p* < 0.01). There were no statistically significant differences in other clinical variables between the two groups. Of note, there was also no significant difference in mortality at 30 days between the 2 groups (15.5% vs. 25%, *p* = 0.326) due to additional 25 deaths in the non-ESRD group after hospital discharge.

**Table 1:** Baseline characteristics of ICH patients with or without ESRD on HD

	Total (*n* = 347)	non-ESRD (*n* = 323)	ESRD on HD (*n* = 24)	*p*-value
Race				0.380
Non-white	196 (56.5%)	185 (57.3%)	11 (45.8%)	
White	151 (43.5%)	138 (42.7%)	13 (54.1%)	
Sex				0.920
Male	213 (61.4%)	199 (60.1%)	14 (58.3%)	
Female	134 (38.6%)	124 (38.4%)	10 (41.7%)	
Age				0.234
< 64 years	169 (48.7%)	154 (47.7%)	15 (62.5%)	
≥ 64 years	178 (51.3%)	169 (52.3%)	9 (37.5%)	
Underlying Conditions				
Hypertension	315 (90.8%)	291 (90.1%)	24 (100%)	0.210
Diabetes Mellitus	138 (39.8%)	119 (36.8%)	19 (79.2%)	**< 0.01**
Hyperlipidemia	129 (37.2%)	116 (35.9%)	13 (24.1%)	0.117
Obesity	79 (22.8%)	77 (23.8%)	2 (8.33%)	0.135
Atrial fibrillation	74 (21.2%)	71 (22.0%)	3 (12.5%)	0.403
History of Smoking	86 (24.8%)	84 (26.0%)	2 (8.33%)	0.091
Chronic Alcohol Intake	40 (11.5%)	39 (12.1%)	1 (4.17%)	0.401
Urine Positive for Meth or Cocaine	31 (8.93%)	31 (9.60%)	0 (0%)	0.223
Anticoagulation use	75 (21.5%)	71 (22.0%)	4 (16.7%)	0.724
Location of hemorrhage				
Subcortical	181 (52.2%)	170 (52.6%)	11 (45.8%)	0.666
Cortical	101 (29.1%)	95 (29.4%)	6 (25.0%)	0.821
Brainstem	25 (7.20%)	24 (7.43%)	1 (4.17%)	0.851
Cerebellum	41 (11.8%)	40 (12.4%)	1 (4.17%)	0.381
IVH	110 (31.7%)	105 (32.5%)	5 (20.8%)	0.338
Initial SBP in ED				0.531
< 180	144 (41.5%)	136 (42.1%)	8 (33.3%)	
≥ 180	203 (58.5%)	187 (57.9%)	16 (66.7%)	
ICH score				0.538
< 3	257 (74.1%)	241 (74.6%)	16 (66.7%)	
≥ 3	90 (25.9%)	82 (25.4%)	8 (33.3%)	
ICU LOS				1.00
< 21 days	330 (95.1%)	307 (95.0%)	23 (95.8%)	
≥ 21 days	17 (4.90%)	16 (4.95%)	1 (4.17%)	
Hospital LOS				0.761
< 21 days	304 (87.6%)	282 (87.3%)	22 (91.7%)	
≥ 21 days	43 (12.4%)	41 (12.7%)	2 (8.3%)	
Mortality				
In-hospital	30 (8.65%)	24 (7.43%)	6 (25.0%)	**< 0.01**
At 30 days	55 (15.9%)	49 (15.2%)	6 (25.0%)	0.326
mRS score				0.135
0–3	139 (47.6%)	134 (48.9%)	5 (27.8%)	
4–5	153 (52.4%)	140 (51.1%)	13 (72.2%)	

ED, emergency department; ESRD, end stage renal disease; HD, hemodialysis; ICH, intracerebral hemorrhage; ICU, intensive care unit; IVH, intraventricular hemorrhage; LOS, length of stay; Meth, methamphetamine; mRS, modified Rankin Scale; SBP, systolic blood pressure

### Propensity score matched cohort characteristics

To construct a control group with reduced bias from the patients without ESRD, propensity score matching of age, gender, race/ethnicity, HTN, DM and tobacco use was performed with a match tolerance of 0.1 and a match ratio 4:1 between the 2 groups. History of chronic kidney disease data was not collected and used for the matching. Two patients in the ESRD group were excluded from the propensity score matching per matching tolerance criteria. As shown in Table 2, after propensity score matching, the ESRD group was associated with significantly lower rate of obesity (9.1% vs. 34.1%, *p* = 0.02) and higher in-hospital mortality (27.3% vs. 8%, *p* = 0.012) than the non-ESRD group. There was no significant difference in other clinical variables. Of note, there was no significant difference in 30-day mortality between the two groups (27.3% vs.18.2%, *p* = 0.340), due to additional 9 deaths after hospital discharge in the non-ESRD group.

**Table 2:** Comparison of ICH patients with versus without ESRD after propensity score matching

	non-ESRD (*n* = 88)	ESRD on HD (*n* = 22)	*p*-value
Race			0.849
Non-white	42 (47.7%)	10 (45.5%)	
White	46 (52.3%)	12 (54.5%)	
Male	51 (58%)	13 (59.1%)	0.990
Age			0.702
< 59 years	40 (45.5%)	11 (50%)	
≥ 59 years	48 (54.5%)	11 (50%)	
Underlying Conditions			
Hypertension	88 (100%)	22 (100%)	1.00
Diabetes Mellitus	66 (75.0%)	18 (81.8%)	0.586
Hyperlipidemia	38 (43.2%)	12 (54.4%)	0.351
Obesity	30 (34.1%)	2 (9.1%)	**0.020**
Atrial fibrillation	23 (25.8%)	3 (13.6%)	0.353
History of Smoking	7 (8.0%)	2 (9.1%)	1.00
Chronic Alcohol Intake	11 (12.5%)	1 (4.5%)	0.453
Urine Positive for Meth or Cocaine	3 (3.4%)	0 (0%)	1.00
Anticoagulation use	17 (19.1%)	4 (18.2%)	1.00
Location of hemorrhage			
Subcortical	57 (64.8%)	10 (45.5%)	0.142
Cortical	19 (21.6%)	6 (27.3%)	0.577
Brainstem	6 (6.8%)	1 (4.5%)	1.00
Cerebellum	10 (11.4%)	1 (4.5%)	0.690
IVH	35 (39.8%)	5 (22.7%)	0.215
Initial SBP in ED			0.921
< 180	31 (35.2%)	8 (36.4%)	
≥ 180	57 (64.8%)	14 (63.6%)	
ICH score			0.841
< 3	58 (65.9%)	14 (63.6%)	
≥ 3	30 (34.1%)	8 (36.4%)	
ICU LOS			0.799
< 21 days	85 (96.6%)	21 (95.5%)	
≥ 21 days	3 (3.4%)	1 (4.5%)	
Hospital LOS			0.487
< 21 days	75 (85.2%)	20 (90.9%)	
≥ 21 days	13 (14.8%)	2 (9.1%)	
Mortality			
In-hospital	7 (8.0%)	6 (27.3%)	**0.012**
At 30 days	16 (18.2%)	6 (27.3%)	0.340
mRS score			0.619
0–3	31 (38.3%)	5 (31.3%)	
4–5	50 (61.7%)	11 (68.7%)	

*Propensity score matching with age, gender, race, HTN, DM and tobacco use, match tolerance 0.1

*ED*,* emergency department; ESRD*,* end stage renal disease; HD*,* hemodialysis; ICH*,* intracerebral hemorrhage; ICU*,* intensive care unit; IVH*,* intraventricular hemorrhage; LOS*,* length of stay; Meth*,* methamphetamine; mRS*,* modified Rankin Scale; SBP*,* systolic blood pressure*

### Clinical characteristics among patients with in-hospital death

Table 3 shows the clinical characteristics of ICH patients with or without ESRD on HD who died during hospitalization. There were no significant differences in severe comorbidities (e.g. dementia, cancer, or other terminal illness), mRS scores at admission, ICH score, active infection or sepsis, and ICU length of stay between the 2 groups. In the ESRD group, the most common cause of in-hospital death was transition to comfort care (83.3%). Only 1 patient died from cardiac arrest (13.6%). In contrast, the common causes of death in the non-ESRD group were comfort care (62.5%) and brain death (25%). There was no statistically significant difference in the causes of death and the time to comfort care decision between the 2 groups, suggesting ESRD on HD per se did not inadvertently influence clinical decision on comfort care.

**Table 3:** Comparison of severe comorbidities and clinical characteristics of the ICH patients who died during hospitalization

	Total (*n* = 30)	Non-ESRD (*n* = 24)	ESRD on HD (*n* = 6)	*p*-value
Pre-existing severe comorbidities				
Dementia	1 (3.33%)	1 (4.17%)	0 (0.00%)	1.00
Cancer	3 (10.0%)	3 (12.5%)	0 (0.00%)	0.879
Other terminal Illness	2 (6.67%)	2 (8.33%)	0 (0.00%)	1.00
mRS score at admission				1.00
0–3	0 (0.00%)	0 (0.00%)	0 (0.00%)	
4–5	30 (100.0%)	24 (100.0%)	6 (100.0%)	
ICH score				1.00
< 3	5 (16.7%)	4 (16.7%)	1 (16.7%)	
≥ 3	25 (83.3%)	20 (83.3%)	5 (83.3%)	
Active infection or sepsis	10 (33.3%)	9 (37.5%)	1 (16.7%)	0.628
ICU LOS (days)				1.00
< 21	29 (96.7%)	23 (95.8%)	6 (100.0%)	
≥ 21	1 (3.33%)	1 (4.35%)	0 (0.00%)	
Transition to comfort care	21 (70.0%)	15 (62.5%)	5 (83.3%)	0.628
Time to comfort care decision (days)	8.5 ± 8.99	7.87 ± 9.82	10.4 ± 6.31	0.518

ESRD, end stage renal disease; HD, hemodialysis; ICH, intracerebral hemorrhage; LOS, length of stay; mRS, modified Rankin Scale

### Univariate logistic regression analysis

The association between individual variables and in-hospital mortality are shown in Table 4. Univariate analysis showed that HTN (OR = 0.18, 95% CI 0.07–0.45, *p* < 0.001), presence of IVH (OR = 3.20, 95% CI 1.37–7.47, *p* = 0.007), and ICH score equal to or greater than 3 (OR = 19.1, 95% CI 6.30–58.0, *p* < 0.001) predicts in-hospital mortality in the non-ESRD group. In contrast, presence of IVH (OR = 13.0, 95% CI 1.20–140, *p* = 0.035), ICH volume greater than or equal to 30 cc (OR = 17.5, 95% CI 1.56–196, *p* = 0.02), and ICH score equal to or greater than 3 (OR = 25.0, 95% CI 2.10–298, *p* = 0.01) were significant predictors of in-hospital mortality in the ESRD group.

**Table 4:** Univariate logistic regression analysis for prediction of in-hospital mortality for the ICH patients with or without ESRD on HD

	Non-ESRD		ESRD on HD	
	Odds ratio (95% CI)	*p* value	Odds ratio (95% CI)	*p* value
Race	0.95 (0.41–2.22)	0.913	2.00 (0.29–13.8)	0.482
Sex	0.79 (0.33–1.90)	0.597	0.63 (0.09–4.33)	0.634
Age	1.08 (0.47–2.50)	0.851	7.86 (0.75–82.1)	0.085
Underlying Disease				
Hypertension	0.18 (0.07–0.45)	**< 0.001**	NA	NA
Diabetes Mellitus	1.25 (0.54–2.90)	0.611	1.43 (0.13–16.0)	0.772
Hyperlipidemia	0.72 (0.29–1.79)	0.476	6.25 (0.60–64.9)	0.125
Obesity	1.07 (0.41–2.80)	0.890	NA	NA
Atrial fibrillation	1.20 (0.46–3.15)	0.711	NA	NA
History of Smoking	0.94 (0.36–2.47)	0.907	3.40 (0.18–64.7)	0.415
Chronic Alcohol Use	2.05 (0.72–5.85)	0.179	NA	NA
Urine positive for Meth or Cocaine	2.02 (0.64–6.33)	0.230	NA	NA
Anticoagulation use	0.69 (0.23–2.10)	0.516	NA	NA
Location of Hemorrhage				
Subcortical	0.42 (0.18–1.02)	0.055	4.00 (0.39–41.5)	0.246
Cortical	1.80 (0.77–4.21)	0.176	0.40 (0.04–4.24)	0.447
Brainstem	1.89 (0.52–6.86)	0.332	NA	NA
Cerebellum	1.46 (0.47–4.52)	0.510	NA	NA
IVH	3.20 (1.37–7.47)	**0.007**	13.0 (1.20–140)	**0.035**
Initial SBP in ED ≥ 180	1.50 (0.62–3.61)	0.368	0.39 (0.06–2.58)	0.325
ICH score ≥ 3	19.1 (6.30–58.0)	**< 0.001**	25.0 (2.10–298)	**0.011**
ICH Volume ≥ 30 cc	NA	NA	17.5 (1.56–196)	**0.020**
ICU LOS ≥ 21 days	0.82 (0.10–6.51)	0.854	NA	NA
Hospital LOS ≥ 21 days	0.98 (0.28–3.45)	0.976	NA	NA

ED, emergency department; ESRD, end stage renal disease; HD, hemodialysis; ICH, intracerebral hemorrhage; ICU, intensive care unit; IVH, intraventricular hemorrhage; LOS, length of stay; Meth, methamphetamine; SBP, systolic blood pressure

### Receiver operating characteristics curve analysis for prediction of 30-day mortality using ICH score

**Fig. 1:**
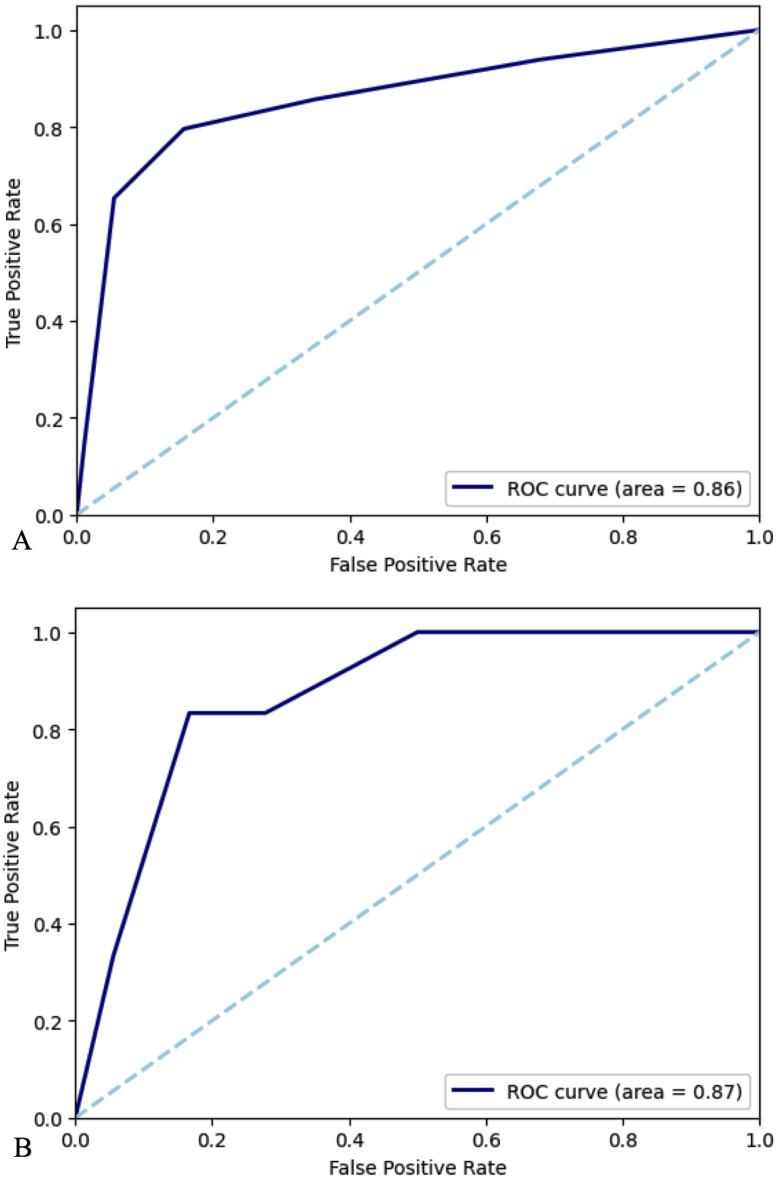
AUC-ROC curves for prediction of 30-day mortality using ICH score. (**A**) non-ESRD group. (**B**) ESRD on HD group

A receiver operating characteristics (ROC) curve analysis was conducted to assess ICH score’s predictive capacity (Table 5; Fig. 1). Among the non-ESRD group, area under the curve (AUC)-ROC for predicting 30-day mortality using the ICH score was 0.858 (95% CI 0.789–0.920), with accuracy 0.415 (95% CI 0.362–0.471), sensitivity 0.939 (95% CI 0.865-1.00), and specificity 0.321 (95% CI 0.264–0.379). Among the ESRD group, AUC-ROC was 0.870 (95% CI 0.690–0.984), with accuracy 0.625 (95% CI 0.417–0.792), sensitivity 1.00 (95% CI 1.00–1.00), and specificity 0.500 (95% CI 0.267–0.737).

**Table 5:** ROC curve analysis for prediction of 30-day mortality using ICH score

	Non-ESRD	ESRD on HD
AUC (95% CI)	0.858 (0.789–0.920)	0.870 (0.690–0.984)
Accuracy (95% CI)	0.415 (0.362–0.471)	0.625 (0.417–0.792)
Sensitivity (95% CI)	0.939 (0.865–1.00)	1.00 (1.00–1.00)
Specificity (95% CI)	0.321 (0.264–0.379)	0.500 (0.267–0.737)

AUC, area under the curve; ESRD, end stage renal disease; HD, hemodialysis; ICH, intracerebral hemorrhage; ROC, receiver operating characteristics

## Discussion

Our study demonstrates that ICH patients with ESRD on HD has a higher in-hospital mortality than patients without ESRD (25% vs. 7.43%). The mortality rate is independently associated with increased ICH score greater than or equal to 3 in both non-ESRD and ESRD cohorts. There was no significant difference in the rate of comfort care or the time to comfort care decision between the two groups, indicating that ESRD on HD status did not inadvertently influence the end-of-life care decision.

After propensity score matching, ICH patients with ESRD on HD had significantly lower rate of obesity and higher in-hospital mortality rate than those without ESRD. This finding suggests a survival benefit from a higher rate of obesity in the non-ESRD group. Obesity has been reported to have a protective effect and survival benefit (obesity paradox) in the patients with ESRD [19, 20] or ICH [21–25]. ESRD on HD patients are more likely to have protein-energy wasting and weight loss [19, 20], and our results indicate that such unique physiological state may contribute to increased mortality among this patient cohort.

Of note, the in-hospital mortality of the ICH patients with ESRD on HD in the current study (25%) were much lower than those reported by other single-center studies (40-67.3%) [7–10]. and a recent multicenter study using the National Inpatient Sample data (37.74%) [26]. The possible explanation for the lower mortality at our comprehensive stroke center is likely attributable to the multidisciplinary care at the dedicated Neuroscience ICU and Stroke Stepdown Unit. While treatment strategies for acute ICH are outlined by AHA/ASA guidelines [18], clinical decisions balancing the risks and benefits of treatment options are critical. This is particularly relevant given conflicting data on the efficacy of certain interventions [27]. ESRD introduces further complexities, risk of hemorrhage expansion, encephalopathy, and infection [28]. In this setting, a dedicated neuro-ICU may provide better care for such complex patients. Our findings reaffirm prior report on reduced mortality rate after ICH in a dedicated Neuroscience ICU [29]. 

Elevated blood pressure is a well-recognized risk factor for ICH among ESRD patients undergoing HD [30, 31]. In our study, a higher initial SBP did not correlate with mortality in both ESRD and non-ESRD groups. These results suggest that while elevated initial SBP is an important risk factor for ICH, it does not influence mortality after ICH [32, 33]. 

Of note we did not collect information on chronic kidney disease (CKD) for the non-ESRD cohort. Given the high prevalence of ICH comorbidities such as HTN, DM, and obesity in the non- ESRD cohort, there is high suspicion that prevalence of CKD is also high. In this context, previous studies have demonstrated that patients with CKD have significantly worse mortality and functional outcome at 90 days and 12 months after ICH [34, 35]. Given such findings, the aim of the current study was to specifically examine the outcome of ICH patients with ESRD on HD versus propensity-score matched controls.

The ICH score is a clinically established grading system for predicting outcomes after ICH. It factors in patient age, ICH volume, and hemorrhage location [13]. Our findings align with other studies that employed the ROC curve analysis for validation of this system, emphasizing the utility of the ICH score for both ESRD and non-ESRD cohorts [36]. Evaluating this system across a spectrum of patient populations, including those with ESRD, is important [37]. 

Nevertheless, it is important to remain cautious when applying predictive scoring systems as the primary determinant of prognosis. Multiple studies have warned of a potential ‘self-fulfilling prophecy’ with these scores, suggesting that they may inadvertently influence clinical decisions, such as early do-not-resuscitate orders, thereby exacerbating patient mortality [38]. In our ESRD group, the common cause of death was comfort care (83.3%). In a recent post hoc analysis of pooled individual patient data from the Clot Lysis: Evaluating Accelerated Resolution of Intraventricular Hemorrhage phase 3 trial (CLEAR-III) and the Minimally Invasive Surgery Plus Alteplase for Intracerebral Hemorrhage Evacuation (MISTIE-III) phase 3 trial, more than 40% of severe ICH and IVH patients with initial poor functional outcome (mRS score 4–5) were shown to have recovered to good functional outcome (mRS score 0–3) by 1 year [14]. This important finding underscores the importance of remaining cognizant of the limitation of any predictive scoring systems. It is essential to provide maximal care to ICH patients with initial poor functional outcome to ensure optimal long-term outcome.

Our study has a few limitations. First, the sample size in the ESRD group was small. Further study with larger sample size is warranted to verify our findings. Second, as a retrospective study, there was no long-term follow-up data. Third, several clinically pertinent variables were not considered in our study (e.g., ventriculostomy or hematoma evacuation). We did not explore their effect due to small sample size.

## Conclusions

In conclusion, ICH patients with ESRD on HD have significantly lower rates of obesity and higher in-hospital mortality than the propensity-score matched non-ESRD controls. Although ICH score ≥ 3 is an independent predictor for poor outcomes in both ESRD and non-ESRD groups, additional study is warranted to investigate long-term functional outcomes among patients with differing underlying clinical characteristics and comorbidities.

## Data Availability

All relevant data are included in manuscript. Our single-institution database utilized in the current study is not publicly available due to privacy constraints relating to the ethical approval. Data sharing was not included as a part of the IRB approval.

## Abbreviations

AUC: Area under the curve
BP: Blood pressure
CKD: Chronic kidney disease
DM: Diabetes mellitus
ED: Emergency department
ESRD: End stage renal disease
GCS: Glasgow coma scale
HD: Hemodialysis
HTN: Hypertension
ICH: Intracerebral hemorrhage
ICU: Intensive care unit
IRB: Institutional review board
IVH: Intraventricular hemorrhage
LOS: Length of stay
mRS: Modified Rankin Scale
ROC: Receiver operating characteristic
SBP: Systolic blood pressure
STROBE: Strengthening the reporting of observational studies in epidemiology

## Declarations

This manuscript has not been published elsewhere and is not under consideration by another journal.
